# Not All Leg Swelling Is Volume Overload: Point‐of‐Care Ultrasound and Sirolimus‐Associated Lymphedema After Combined Heart‐Kidney Transplant

**DOI:** 10.1002/ccr3.72717

**Published:** 2026-05-14

**Authors:** Rebecca Boyle, Neev Patel

**Affiliations:** ^1^ Stanford Hospital and Clinics, Department of Nephrology Palo Alto California USA; ^2^ Stanford University, Department of Nephrology Palo Alto California USA

**Keywords:** case report, heart‐kidney transplant, lymphedema, point‐of‐care ultrasound, sirolimus, volume assessment

## Abstract

A 65‐year‐old man, one year post combined heart‐kidney transplant, presented with acute kidney injury, dyspnea, and progressive leg edema unresponsive to escalating diuretics. Despite suspicion for volume overload, multi‐organ point‐of‐care ultrasound (POCUS) revealed bilateral A‐lines without B‐lines or effusions, a small collapsible inferior vena cava, and preserved biventricular function, all inconsistent with cardiogenic congestion. The edema was attributed to sirolimus‐associated lymphedema. Diuretic cessation and transition from sirolimus to everolimus improved renal function. This case illustrates the utility of integrated multi‐organ POCUS in challenging assumptions about volume status and underscores the importance of contextualizing findings across multiple sonographic windows.

## Key Clinical Message

Not all edema reflects hypervolemia. In complex transplant recipients, integrated multi‐organ point‐of‐care ultrasound can differentiate systemic congestion from non‐cardiac causes such as drug‐induced lymphedema, preventing diagnostic anchoring and iatrogenic harm from unnecessary diuresis.

## Graphical abstract

Multi‐organ point‐of‐care ultrasound in a combined heart‐kidney transplant recipient with refractory leg edema demonstrated bilateral A‐lines, a collapsible inferior vena cava, and preserved biventricular function, excluding cardiogenic congestion. Right heart catheterization confirmed a low‐filling‐pressure, high‐output state. Sirolimus‐associated lymphedema was diagnosed; edema resolved after transition off mTOR inhibitor therapy.

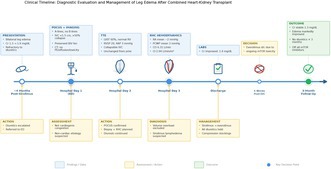

## 1 Introduction

Lower‐extremity edema in solid‐organ transplant recipients is commonly attributed to volume overload, prompting empiric diuretic escalation that can precipitate acute kidney injury and graft compromise. Multi‐organ point‐of‐care ultrasound (POCUS), which integrates lung, inferior vena cava, and focused cardiac views, provides a portable bedside framework for volume assessment, and concordant findings across modalities approximate the accuracy of invasive hemodynamics [1, 2]. We describe a patient one year after combined heart‐kidney transplantation who presented with refractory lower‐extremity edema and acute kidney injury, in whom integrated multi‐organ POCUS guided the bedside evaluation and informed subsequent management.

## 2 Case History and Examination

A 65‐year‐old man presented to the emergency department with progressive bilateral lower extremity edema, acute kidney injury, and exertional dyspnea refractory to escalating outpatient diuretics. He had undergone a simultaneous heart‐kidney transplant one year earlier for cardiac and renal sarcoidosis. His post‐transplant course was complicated by recurrent leukopenia, indeterminate cardiac allograft biopsies, and granulomatous tubulointerstitial nephritis consistent with recurrent sarcoidosis (diagnosed on kidney allograft biopsy three months prior, treated with pulse steroids and methotrexate). Persistent leukopenia and concern for cardiac allograft vasculopathy had prompted transition from mycophenolate to sirolimus (target trough 5–8 ng/mL) four months before presentation, while continuing tacrolimus and prednisone 10 mg daily. Family and social history were non‐contributory.

Following sirolimus initiation, the patient developed progressive bilateral lower extremity edema with serum creatinine rising from a baseline of 1.3 to 1.9 mg/dL. Outpatient diuretics were escalated aggressively: Furosemide 80 mg twice daily was trialed without improvement, then transitioned to bumetanide 2 mg twice daily with hydrochlorothiazide 12.5 mg daily, and ultimately increased to bumetanide 3 mg twice daily with hydrochlorothiazide, all without a meaningful reduction in edema. The patient reported approximately 5 pounds of weight loss over the four weeks before presentation, likely reflecting the effects of escalating diuretics. Both the outpatient cardiology and nephrology teams suspected volume overload, prompting referral to the emergency department.

On presentation, vital signs were stable: Temperature 36.8°C, BP 153/81 mmHg, HR 86 bpm, and SpO2 96% on room air. He reported exertional dyspnea, lightheadedness, and dizziness but denied orthopnea, paroxysmal nocturnal dyspnea, cough, chest pain, and abdominal pain. Examination revealed asymmetric pitting edema of both lower extremities with warm extremities, no jugular venous distension, clear lung fields, regular cardiac rhythm without murmurs, and no ascites or hepatomegaly. Admission laboratories were notable for serum creatinine 1.9 mg/dL (baseline 1.3) and uric acid 11.1 mg/dL, consistent with volume depletion in the setting of aggressive diuresis.

## 3 Differential Diagnosis, Investigations, and Treatment

**FIGURE 1:**
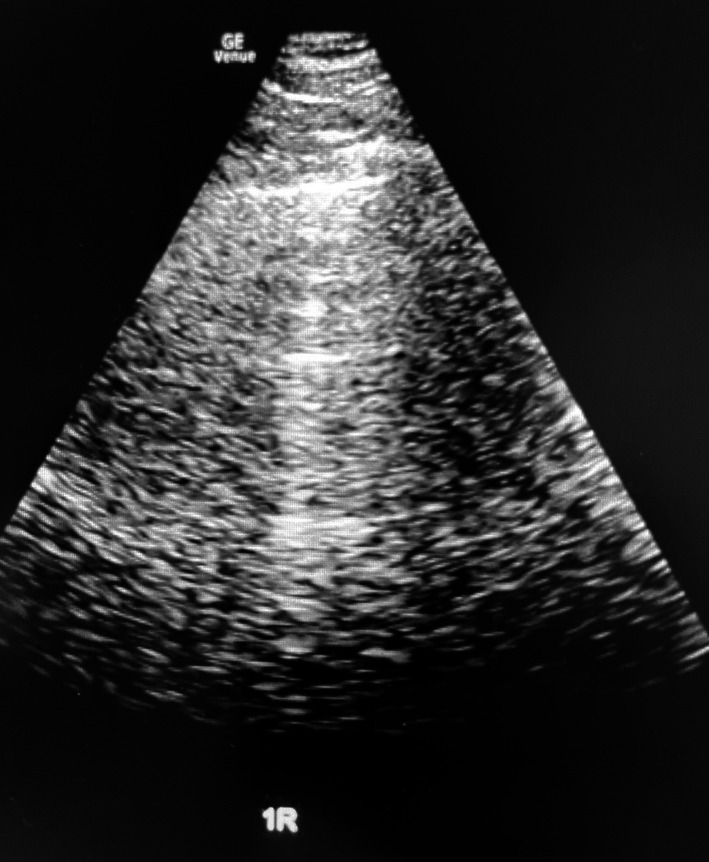
Lung ultrasound image from the right anterior chest showing horizontal A‐lines with absence of B‐lines or pleural effusion, consistent with the absence of interstitial pulmonary edema.

**FIGURE 2:**
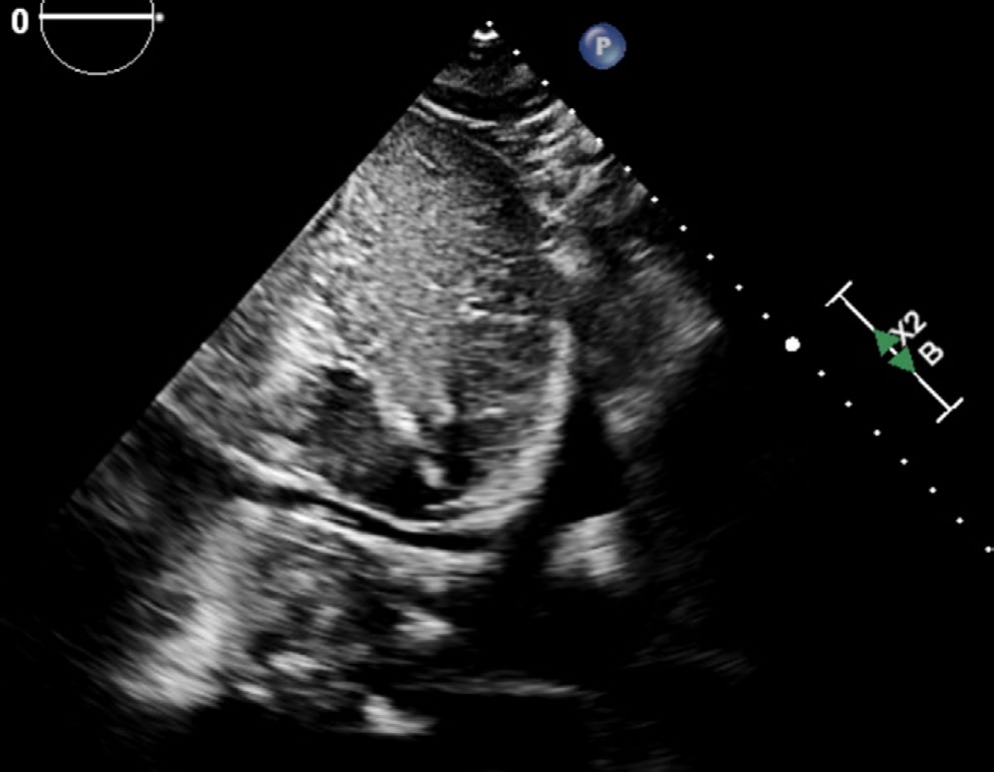
Subcostal long‐axis view of the inferior vena cava (IVC) in a patient with a transplanted heart. The IVC measures less than 2 cm in diameter and demonstrates greater than 50% collapsibility with respiration, suggesting a right atrial pressure less than 5 mmHg.

**FIGURE 3:**
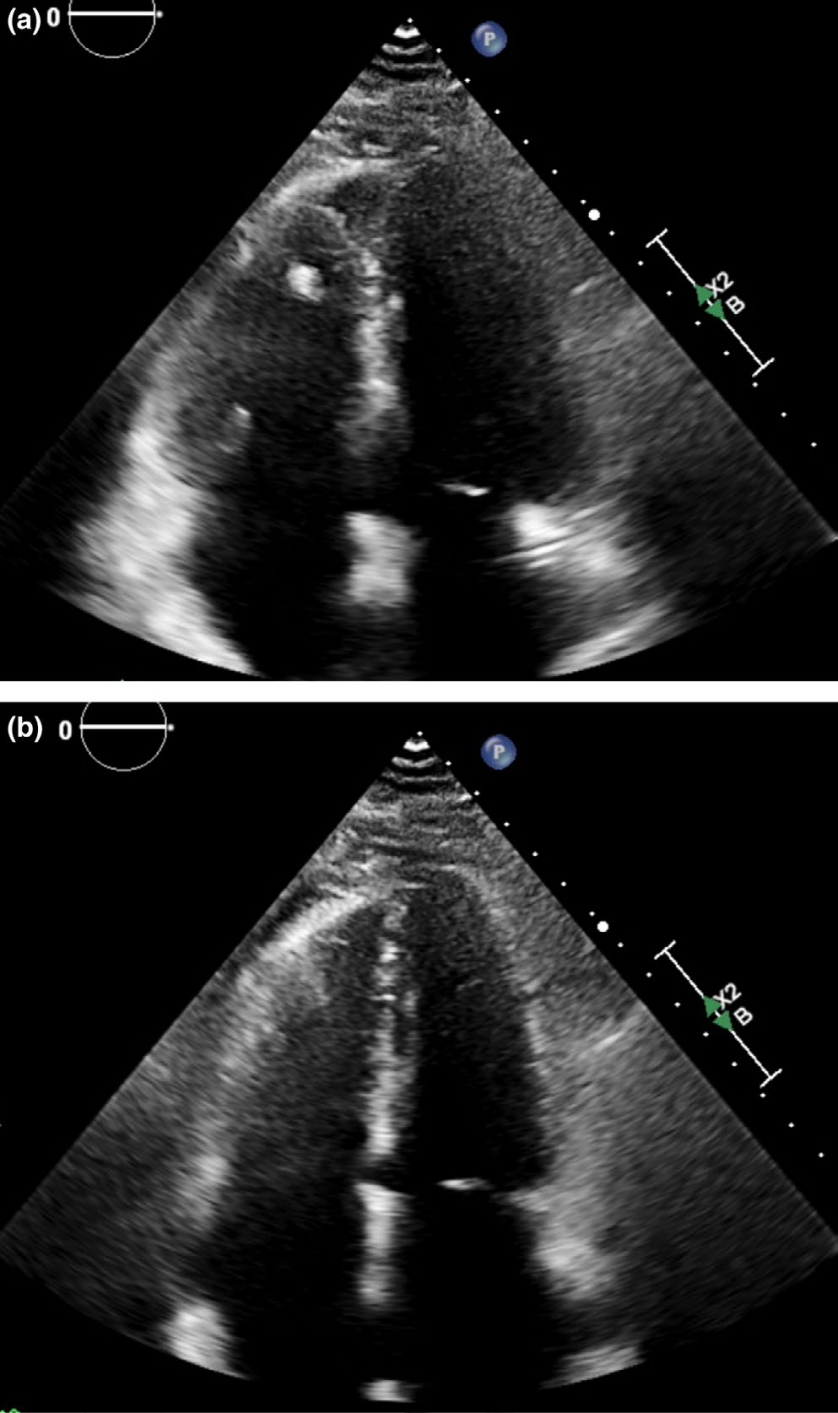
(a) Apical four‐chamber view during end‐diastole showing normal right and left ventricular chamber sizes and geometry without chamber dilation or septal flattening, consistent with the absence of right ventricular volume or pressure overload. (b) Apical four‐chamber view during end‐systole showing preserved systolic contraction without regional wall motion abnormality.

Multi‐organ point‐of‐care ultrasound (POCUS) was performed by the nephrology team in the emergency department. Lung ultrasound revealed bilateral A‐lines without B‐lines or pleural effusions, consistent with absence of pulmonary edema (Figure 1) [3]. The inferior vena cava (IVC) measured less than 1.5 cm with > 90% inspiratory collapsibility, suggesting low right atrial pressure (RAP) (Figure 2). Focused cardiac ultrasound from the apical four‐chamber view showed normal biventricular chamber sizes, preserved systolic function, and no septal flattening (Figure 3a,b), without right ventricular dilation or dysfunction. The overall assessment was inconsistent with cardiogenic congestion, prompting the nephrology team to suspect sirolimus‐associated lymphedema and broaden the differential to non‐cardiac causes of leg swelling. A non‐contrast chest CT obtained to evaluate for sirolimus pulmonary toxicity showed no acute parenchymal abnormality.

Despite these findings, the heart transplant team initiated intravenous bumetanide on hospital day 1, driven by the significant leg swelling. Formal transthoracic echocardiography (TTE) on hospital day 2 confirmed the POCUS findings: Preserved biventricular function (LVEF 60%), estimated RAP 3 mmHg, and a collapsible IVC, unchanged from a prior TTE two months earlier. A protocol endomyocardial biopsy, already scheduled as part of 12‐month post‐transplant surveillance, was advanced to hospital day 3, given the acute presentation. Right heart catheterization (RHC) was performed concurrently, as the biopsy was already indicated, and concurrent catheterization added minimal procedural risk while providing definitive hemodynamic data. In a transplanted heart with altered physiology and prior indeterminate biopsies, invasive hemodynamics offered certainty that surrogate echocardiographic indices could not. Hemodynamics revealed RA pressure 2/0 (mean−2) mmHg, PA pressure 22/5 (mean 12) mmHg, and PCWP 6/4 (mean 3) mmHg, with Fick‐derived cardiac output of 6.3 L/min and cardiac index 2.9 L/min/m^2^, confirming a low‐filling‐pressure, high‐output state consistent with the POCUS assessment.

Only after RHC confirmed the absence of volume overload did the heart transplant team consider alternative etiologies. Given the temporal relationship between sirolimus initiation and symptom onset, sirolimus‐associated lymphedema was favored. Abdominal ultrasound, performed to exclude hepatic congestion or venous outflow obstruction as contributors to leg swelling, showed no acute abnormalities and no signs of elevated central venous pressure. All diuretics were held, and the patient was managed with daily weight monitoring and as‐needed diuresis, which was not required during the remainder of hospitalization. Sirolimus was discontinued and replaced with everolimus (target trough 3–8 ng/mL). Compression stockings were prescribed. Serum creatinine improved from 1.9 to 1.4 mg/dL at discharge (weight 98.7 kg), and the patient was referred to a multidisciplinary lymphedema clinic.

## 4 Conclusion and Results (Outcome and Follow‐Up)

**FIGURE 4:**
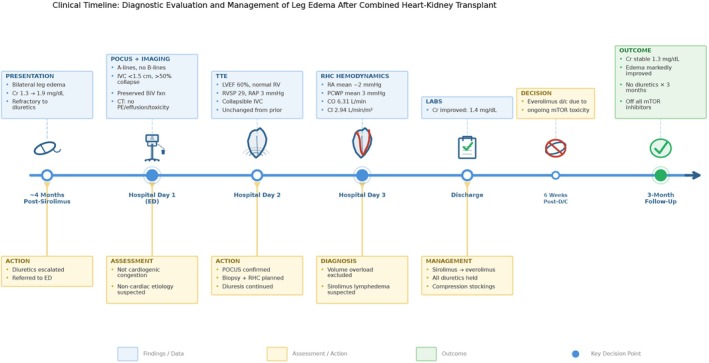
Clinical timeline summarizing the diagnostic evaluation and management of leg swelling after a combined heart‐kidney transplant. Upper panels (blue) display findings from point‐of‐care ultrasound, transthoracic echocardiography, right heart catheterization, and laboratory data at each time point. Lower panels (yellow) show the corresponding clinical assessments and management decisions. The timeline illustrates the progression from initial presentation with bilateral leg edema refractory to escalating diuretics through systematic exclusion of cardiogenic congestion via multi‐organ POCUS and invasive hemodynamics, leading to the diagnosis of sirolimus‐associated lymphedema and subsequent immunosuppression modification. Filled blue circles denote key decision points. BiV, biventricular; CI, cardiac index; CO, cardiac output; Cr, creatinine; CT, computed tomography; d/c, discontinued; ED, emergency department; IVC, inferior vena cava; LVEF, left ventricular ejection fraction; mTOR, mechanistic target of rapamycin; PCWP, pulmonary capillary wedge pressure; PE, pulmonary embolism; POCUS, point‐of‐care ultrasound; RA, right atrial; RAP, right atrial pressure; RHC, right heart catheterization; RV, right ventricle; RVSP, right ventricular systolic pressure; TTE, transthoracic echocardiography.

The patient required no diuretic therapy for three months after discharge. Everolimus was discontinued approximately six weeks after initiation due to persistent concerns regarding mTOR inhibitor‐related adverse effects, including persistent leg swelling. At the three‐month follow‐up, lower extremity swelling had improved dramatically, though mild residual edema in the right foot and ankle persisted. Serum creatinine had stabilized at 1.3 mg/dL without signs of allograft rejection. The complete clinical course, diagnostic evaluation, and management decisions are summarized in Figure 4.

In conclusion, integrated multi‐organ POCUS offers a practical, bedside framework for individualized volume assessment that can prevent diagnostic anchoring and iatrogenic harm in transplant recipients. Prospective studies validating structured POCUS protocols, including venous excess ultrasonography (VExUS) and other objective measures of congestion in transplant populations, are needed to advance evidence‐based volume management in this complex patient group.

## 5 Discussion

This case illustrates the diagnostic value of integrated multi‐organ POCUS in evaluating volume status in a complex combined heart‐kidney transplant patient while highlighting the limitations of isolated IVC assessment. The convergence of bilateral A‐lines, a near‐completely collapsible IVC (> 90% inspiratory collapse), and preserved cardiac function effectively excluded cardiogenic congestion. The absence of pulmonary edema on lung ultrasound was particularly instructive, providing objective evidence of normal extravascular lung water despite the patient's dyspnea and clinical suspicion for volume overload.

The diagnostic performance of commonly used bedside findings for volume assessment is summarized in Table 1; no isolated physical‐examination sign or single sonographic measurement has both high sensitivity and high specificity for cardiopulmonary congestion, which is the central rationale for an integrated, multi‐organ approach. The IVC is subject to well‐documented limitations: Respiratory variation does not reliably predict fluid responsiveness in all populations and is influenced by ventilation status, abdominal compliance, and patient cooperation [11, 12, 13, 14]. IVC dimensions alone cannot distinguish true hypovolemia from distributive processes or increased capillary permeability. In this patient, convergent findings across clinical (warm extremities, stable hemodynamics, no orthostasis), biochemical (AKI with elevated uric acid), and sonographic (clear lungs, small IVC, preserved cardiac function) domains supported hypovolemia. RHC provided definitive hemodynamic confirmation. The negative RAP and markedly reduced PCWP (3 mmHg) excluded volume overload, while the elevated cardiac output (6.3 L/min) further supported a non‐cardiogenic, distributive process consistent with the bedside POCUS assessment.

**TABLE 1:** Diagnostic performance of bedside findings used for volume assessment and cardiopulmonary congestion.

Finding	Reference Target	Study Population (n)	Sensitivity, % (95% CI)	Specificity, % (95% CI)	References
Physical examination					
Peripheral edema (isolated sign)	Heart failure (primary care)	IPD meta‐analysis	53	72	Mant, 2009 [4]
Jugular venous distension, rest or inducible	PCWP ≥ 18 mmHg	52	81	80	Butman, 1993 [5]
Third heart sound (S_3_)	Heart failure	5614 (19 studies)	23 (15–33)	94 (82–98)	Dao, 2022 [6]
Rales, peripheral edema, and elevated JVP (all three)	PCWP ≥ 22 mmHg	43	58	100	Stevenson, 1989 [7]
Lung ultrasound					
B‐lines (≥ 3 in ≥ 2 bilateral zones), integrated LUS algorithm	Acute decompensated heart failure	1005	97	97	Pivetta, 2015 [8]
B‐lines (meta‐analysis)	Acute heart failure	Meta‐analysis	85	93	Martindale, 2016 [1]
B‐profile (BLUE protocol)	Cardiogenic pulmonary edema	260	97	95	Lichtenstein, 2008 [2]
A‐profile (BLUE protocol)	Non‐edematous lung (asthma, COPD)	260	89	97	Lichtenstein, 2008 [2]
Inferior vena cava					
IVC diameter ≥ 2.0 cm	RAP > 10 mmHg	102	73	85	Brennan, 2007 [9]
IVC collapsibility > 40%	RAP < 10 mmHg	102	73	84	Brennan, 2007 [9]
Caval index ≥ 50%	CVP < 8 mmHg	72	91 (71–99)	94 (84–99)	Nagdev, 2010 [10]

*Note:* Values are reported as point estimates; 95% confidence intervals are shown in parentheses where published. Diagnostic performance varies with the reference target (e.g., PCWP ≥ 18 vs. ≥ 22 mmHg; RAP cutoffs), the population studied (chronic heart failure, undifferentiated dyspnea, or acute respiratory failure), and pretest probability. No single bedside finding simultaneously achieves both high sensitivity and high specificity, which supports the integration of complementary findings across multiple sonographic windows.

Abbreviations: BLUE, Bedside Lung Ultrasound in Emergency; COPD, chronic obstructive pulmonary disease; CVP, central venous pressure; IPD, individual patient data; IVC, inferior vena cava; JVP, jugular venous pressure; LUS, lung ultrasound; PCWP, pulmonary capillary wedge pressure; RAP, right atrial pressure.

POCUS offers distinct advantages in transplant medicine: It is portable, repeatable, radiation‐free, and enables real‐time integration of sonographic findings with clinical context at the bedside. In transplant recipients, where fluid status is dynamic and influenced by calcineurin inhibitor nephrotoxicity, variable graft function, and polypharmacy, rapid multi‐organ volume assessment represents a meaningful advance over static clinical evaluation. However, POCUS is operator‐dependent, and altered cardiac anatomy (denervated heart, surgical changes), chronic medication effects, and atypical presentations may limit the applicability of reference values derived from non‐transplant cohorts. Prospective validation of integrated POCUS protocols in transplant recipients is needed.

The temporal relationship between sirolimus initiation and symptom onset implicated sirolimus‐associated lymphedema. This recognized, but underappreciated, complication of mTOR inhibitor therapy has been documented in both renal and cardiac transplant populations [15, 16, 17, 18, 19]. Proposed mechanisms include direct lymphatic endothelial injury, impaired lymphangiogenesis, and altered lymphatic contractility [15, 16]. The diagnosis requires a high index of suspicion and exclusion of more common etiologies, including volume overload, venous insufficiency, and hypoalbuminemia. Improvement after transition to everolimus supports the diagnosis, though concurrent diuretic de‐escalation and improved graft function preclude definitive attribution. Notably, following complete discontinuation of mTOR inhibitor therapy, lower extremity edema decreased but did not fully resolve within two weeks, ultimately prompting cessation of everolimus and further supporting a class‐related, drug‐mediated etiology.

Several limitations warrant mention. This is a single‐case observation that cannot be generalized without further study. The diagnosis of sirolimus‐associated lymphedema was clinical, based on temporal association with drug initiation and improvement after substitution; histological confirmation was not obtained, and alternative causes such as chronic venous insufficiency or subclinical graft dysfunction were not formally excluded. The hemodynamic confirmation available here through concurrent RHC may not be feasible in many clinical settings, limiting the generalizability of the diagnostic pathway described. Nevertheless, the concordance between bedside POCUS and invasive hemodynamics validates the ultrasound‐driven approach, which may be particularly valuable in settings where RHC is not readily available or in resource‐limited environments where clinical decision‐making relies predominantly on non‐invasive assessment.

## Author Contributions

**Rebecca Boyle:** conceptualization, data curation, visualization, writing – original draft. **Neev Patel:** conceptualization, resources, software, visualization, writing – review and editing.

## Funding

The authors have nothing to report.

## Disclosure

Patient Perspective: The patient was not available to provide a written perspective for this report.

## Ethics Statement

This is a retrospective, de‐identified clinical case with no intervention outside standard care.

## Consent

Written informed consent was obtained from the patient for publication of this case report and accompanying images.

## Conflicts of Interest

The authors declare no conflicts of interest.

## Data Availability

Data sharing is not applicable to this article as no datasets were generated or analysed; all clinically relevant findings are reported in full within the manuscript, and de‐identified patient information is included with the patient's written consent.
